# A low cost, superhydrophobic and superoleophilic hybrid kaolin-based hollow fibre membrane (KHFM) for efficient adsorption–separation of oil removal from water

**DOI:** 10.1039/c7ra13206a

**Published:** 2018-01-15

**Authors:** Siti Khadijah Hubadillah, Preven Kumar, Mohd Hafiz Dzarfan Othman, A. F. Ismail, Mukhlis A. Rahman, Juhana Jaafar

**Affiliations:** Advanced Membrane Technology Research Centre (AMTEC), Faculty of Chemical and Energy Engineering, Universiti Teknologi Malaysia 81310 Skudai Johor Malaysia dzarfan@utm.my hafiz@petroleum.utm.my

## Abstract

Inspired by the lotus leaf surface structure, which possesses a hydrophobicity behaviour, a low cost, high performance superhydrophobic and superoleophilic kaolin hollow fibre membrane (KHFM) was obtained by a simple sol–gel grafted method using tetraethoxysilane (TEOS) and methyltriethoxysilane (MTES) for oil removal from water. The KHFM was grafted at various grafting times ranging from 1 to 5 coating cycles. Prior to the calcination process at 400 °C, the grafted KHFM was dried in an oven at 100 °C for 1 hour for each grafting coating cycle. The grafting process efficiency was measured by the contact angle of water and hexane. Scanning electron microscopy (SEM) and Atomic Force Microscopy (AFM) were used to study the morphology and surface roughness, respectively, of the grafted KHFM. The oil removal was conducted by using the homogeneous mixture of hexane and water. The highest hydrophobicity and oleophilicity was obtained for the KHFM grafted at 2 coating cycles with a contact angle value equal to 157° and 0°, respectively. In fact, the mechanical strength of KHFM was also improved from 16.21 MPa to 72.33 MPa after grafting. In terms of performance, KHFM grafted for 2 coating cycles obtained an almost 99.9% absorption of oil. Thereby, KHFMs were assembled into a module for a filtration study. A high oil flux of 102 L m^−2^ h^−1^ was obtained for superhydrophobic and superoleophilic KHFM with 2 grafting coating cycles of 2, and this result is in agreement with the trend of the adsorption result.

## Abstract 2

This paper outlines a low cost, high performance superhydrophobic/superoleophilic KHFM through a simple sol–gel grafted method using TEOS and MTES for efficient adsorption–separation of oil removal from water.

## Graphical abstract

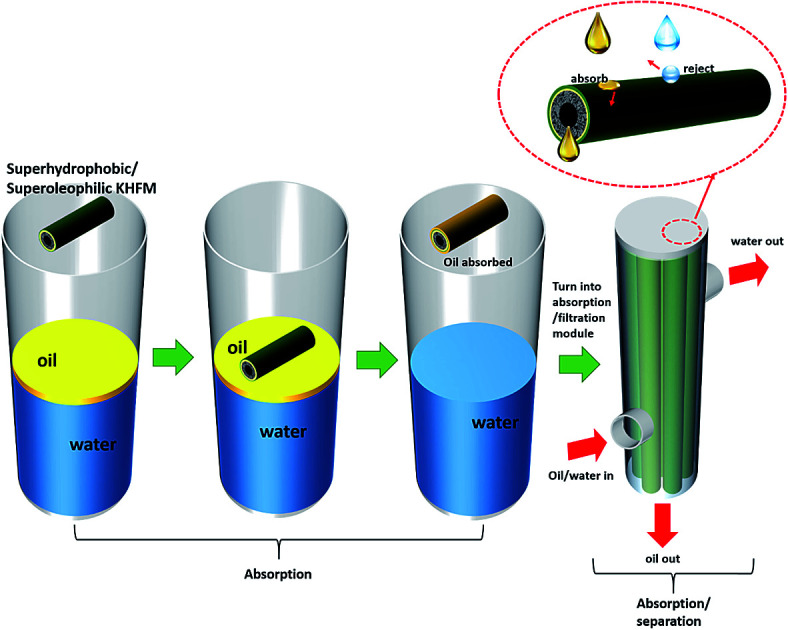

## 1. Introduction

The 19th century was an epoch of great change and very mushrooming industrialization. The iron and steel industry proliferated new construction materials, the railroads connected around the world and the discovery of oil bestowed a new source of fuel. The discovery of the Spindle top geyser in 1901 drove a huge growth in the oil industry. Within a few years, more than 1500 oil companies had been chartered and oil became the dominant fuel of the 21st century and an integral part of many countries' economies.^1^ Consequently, there has been an increasing concern about the environmental risks that have arisen from the probability of oil spill accidents and increasing industrial oily wastewater.^2^ Moreover, increasing volumes of crude oil production, transportation and storage have increased the risks of spills to marine and freshwater environments. In recent decades, high-profile spills are numerous, causing not only loss of the energy resource but also significant injuries to the environment and ecosystems.^3^ Therefore, an urgent need is to find a method to separate these oils from water.

Among all the methods, the most common way is to use adsorbent materials for oil and water separation.^4^ In 2002, Nishi *et al.*^5^ investigated the sorption kinetics of oil into porous carbons. They found that the sorption kinetic is dependent on the density and pore structure of porous carbon. Feng *et al.*^6^ studied the oil absorption on a superhydrophobic cost effective cellulose aerogel derived from paper waste. A very stable superhydrophobic (for over five months) was obtained with an absorption capacity of 95 g g^−1^. However, the absorption alone shows some drawbacks, including less recoverability and reusability.

Recently, the development of superhydrophobic and superoleophilic grafts on porous substrates for oil removal from water has been attracting increasing attention. Zhou *et al.*^7^ developed superhydrophobic and superoleophilic sponges by a simple vapour phase deposition process and found the absorption capacity of the sponge increased up to 20 times. Xu *et al.*^8^ used cotton fabrics as a substrate for a superhydrophobic study using silica oxide (SiO_2_) and zinc oxide (ZnO). Consequently, copper meshes have also been used extensively for superhydrophobic and superoleophilic porous substrates as they offer a high mechanical strength.^9–11^ To date, more studies have focused on superhydrophobic and superoleophilic grafts on a stainless steel mesh have been focused due to their anti-chemical erosion, anti-heat ageing and easy obtainability.^12–16^ However, stainless steel is expensive and its industrial application is limited to 500$ per m^2^ to 1000$ per m^2^.^17^ The other disadvantage of stainless steel is the lack of flexibility which limits possible shapes to achieve a high surface area. In view of efficiency, these studies focused on an adsorption method only for which further study on separation is still very much required to complete the process.

Nowadays, a ceramic membrane is introduced as an innovative product and has received extensive attention in water treatment, including oil and water separation. A fabrication of a tubular ceramic membrane consisting of kaolin, quartz, ball clay, pyrophyllite and feldspar has been studied by Kumar *et al.*^18^ towards microfiltration of synthetic oily wastewater and 99.98% of oil rejection obtained. Madaeni *et al.*^19^ obtained a 100% rejection of oil from petrochemical oily wastewater using a ceramic membrane from γ-Al_2_O_3_. Among all the ceramic materials for ceramic membrane fabrication, kaolin is one of the most used and applied due to its cost effectiveness, porous structure, low plasticity and high refractory properties.^20–22^

Our recent work demonstrated the successful fabrication of a low cost superhydrophilic ceramic membrane from kaolin in a hollow fibre configuration, being called a kaolin hollow fibre membrane (KHFM), for oily wastewater separation *via* a combined phase inversion and sintering technique.^23^ A 100% of oil rejection was obtained due to its smaller pore size at a higher sintering temperature of 1500 °C. Conversely, the smaller pore size exhibited a lower flux with a value of less than 50 L m^−2^ h^−1^ although having a 33° contact angle value. Increasing the pore size will lead to a flux enhancement; however, *vice versa* for the oil rejection performance. Taking advantage of the porous structure offered by KHFM sintered at 1300 °C with a porosity and average pore size of 44.2% and 1.42 μm, herein we report a novel preparation of low cost superhydrophobic and superoleophilic KHFM for the adsorption–separation process of oil and water. KHFM is grafted at various grafting times ranging from 1 to 5 coating cycles and characterized in term of morphological study, strength, wettability and adsorption–separation performance.

## 2. Experimental

### 2.1 Materials

Kaolin hollow fibre membrane (KHFM) prepared *via* phase inversion and sintered at 1300 °C (from our previous study) was used and applied as the substrate.^23^ Tetraethoxysilane (TEOS) (98%), methyltriethoxysilane (MTES) (98%), ammonia (NH_3_ 25%), absolute ethanol (EtOH, 99.5%), and distilled water (H_2_O) were used throughout this work. The chemicals were used without any modification and further purification.

### 2.2 Preparation of silica sol–gel solution

**Fig. 1:**
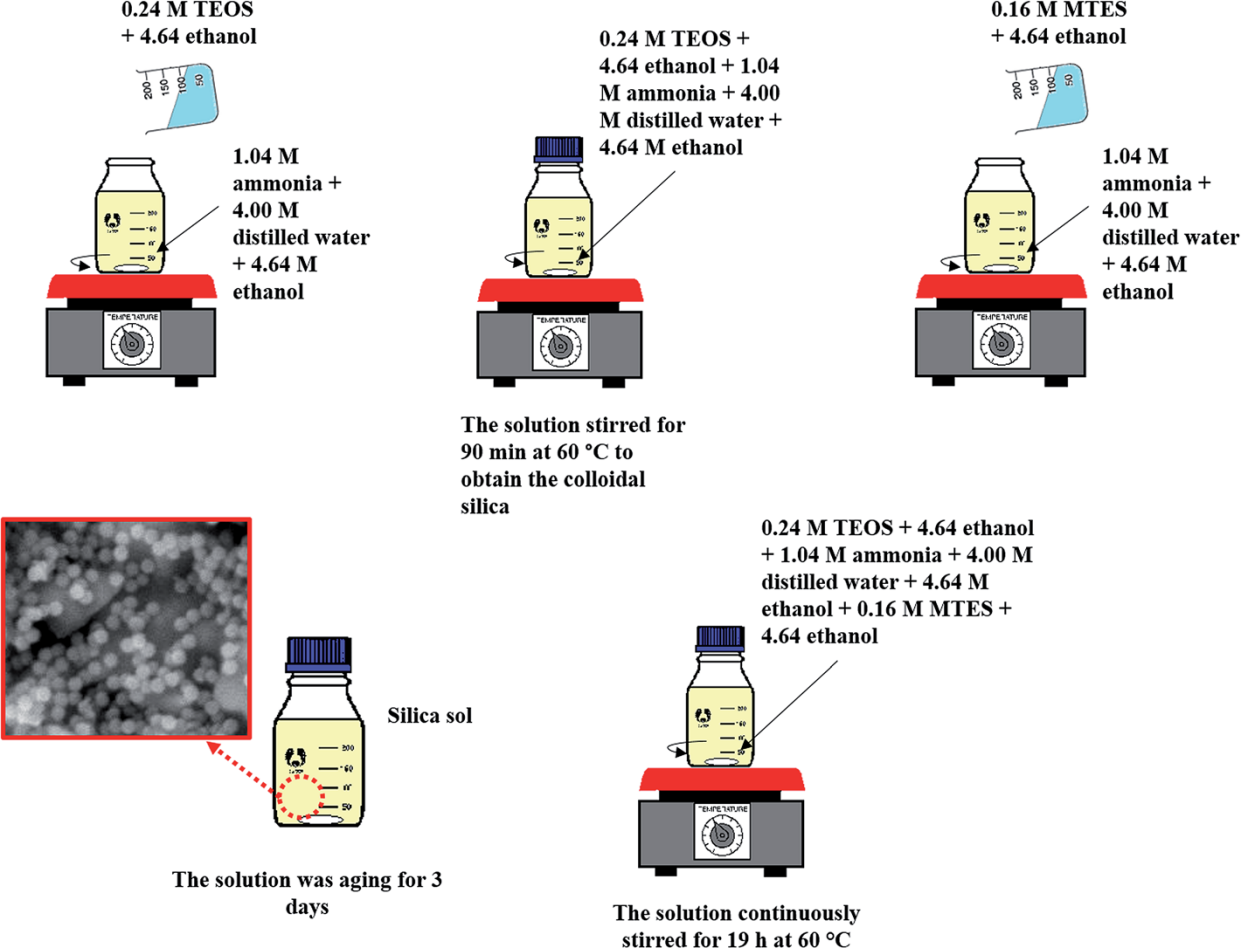
Schematic diagram for the preparation of the silica sol–gel solution.

**Fig. 2:**
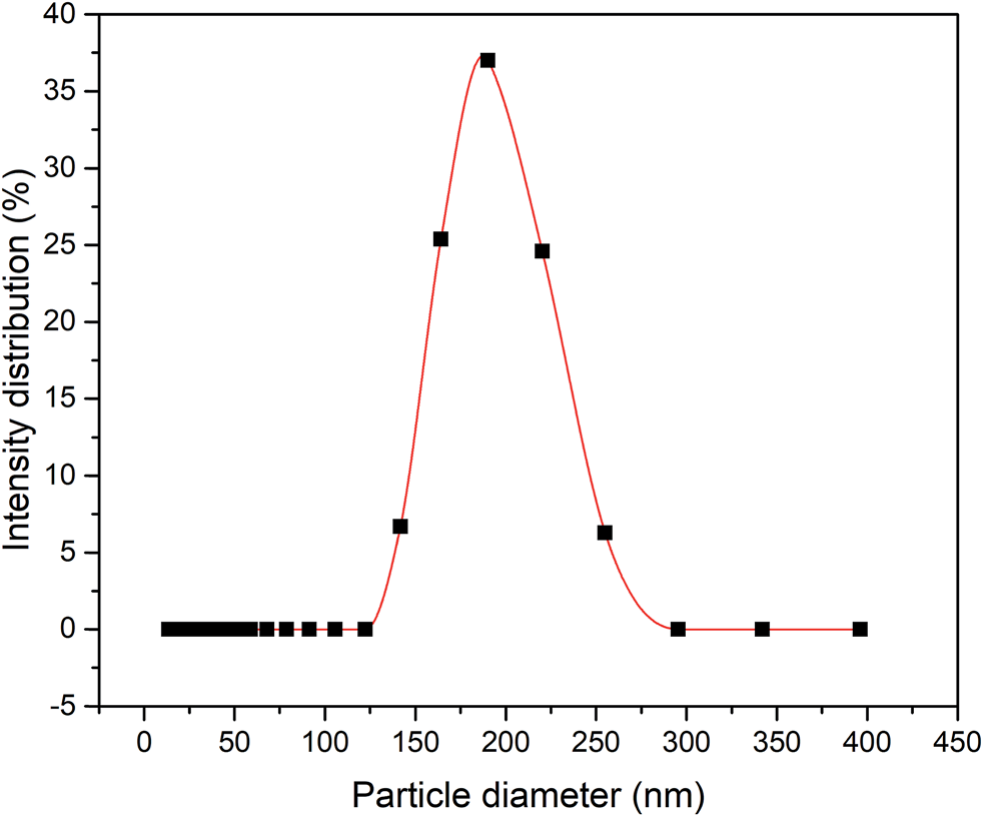
Particle size distribution of the methylated silica sol prepared through a modified Stöber method.

Fig. 1 illustrates the method used in preparing the silica sol–gel through a modified Stöber method, which was adapted by Yang *et al.*'s work.^16^ Firstly, 0.24 M TEOS and 4.64 M ethanol were added to the mixture of 1.04 M ammonia, 4.00 M H_2_O and 4.64 M ethanol. The mixture was then allowed to react for 90 min at 60 °C to obtain the colloidal silica. Consequently, the exact amount of 0.16 M MTES and 4.64 M ethanol was then added to the solutions to create CH_3_ bonds and produce a methylated silica sol. Thereafter, the solutions were continuously stirred for 19 h at 60 °C. Then, the solution was aged for exactly 3 days under ambient temperature and pressure to allow gelation to occur. Fig. 2 presents the particle size of the methylated silica sol measured using a particle size analyser (Zetasizer Version 7.11, Malvern, UK).

### 2.3 Preparation of superhydrophobic/superoleophilic KHFM

**Fig. 3:**
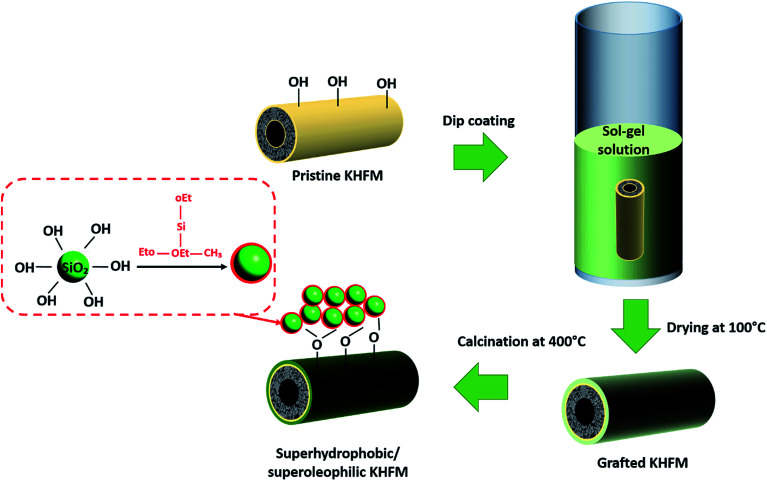
Schematic illustration of the procedure for the preparation of superhydrophobic/superoleophilic KHFM.

Prior to the grafting process, the ceramic membrane first went through a hydrolysis process by immersion of the KHFM in a solution of 2 : 1 ethanol : distilled water for 24 hours. After drying at 100 °C overnight, the KHFM was dipped into the sol gel solutions for 30 minutes as illustrated in Fig. 3. The grafted ceramic membrane was then dried at 100 °C for 1 hour. This step was repeated for 2 coating cycles, 3 coating cycles, 4 coating cycles and 5 coating cycles, respectively, for the effect of grafting time. The dried grafted KHFM was calcined at 400 °C for 2 hours inside the furnace to obtain the final product.

### 2.4 Characterizations and performance of KHFM

The surface morphologies of KHFM before and after grafting were observed by a scanning electron microscope (SEM: JSM-6010LA; Jeol, Japan) operating at 30 kV and 10 000 magnification. The contact angle and tilt angle measurements were performed with an optical contact angle machine (OCA 40 Micro, Dataphysics, Germany). The wettabilities of the KHFM before and after grafting were determined by measuring the contact angle of water and hexane. The hydrophobicity and oleophilicity were measured for 5 coating cycles with 0.5 μL of water and 0.5 μL of hexane with a 0.5 μL s^−1^ drop time. The surface roughness of the KHFM was analysed by atomic force microscopy (AFM: XE-100; Park system, Korea). The scanning scale was 10 μm × 10 μm. The mechanical strengths of KHFM before and after grafting were examined by a three-point bending test using an Instron Model 5544 tensile tester provided with a load cell of 1 kN. KHFMs were fixed on the sample holder with a 5 cm distance. The bending strength (*σ*_F_) is calculated using the following equation:^24^1
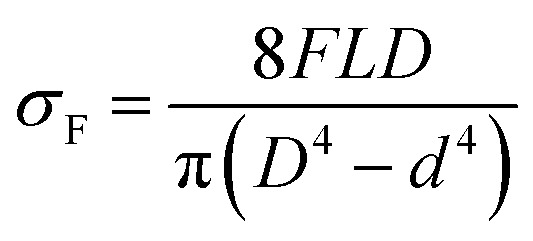
where *F* is the force measured at the fracture point of the KHFM, *L* is the span (5 cm), and *D* and *d* are the outer and inner diameters of the KHFM, respectively. Fourier transform infrared spectroscopy (FTIR: Spectrum 100, PerkinElmer, Waltham, MA) was used to study qualitatively the methyl groups grafted on the KHFM.

### 2.5 Oil removal from water through absorption and separation

The study for oil removal was divided into two; (1) to study the oil absorption capacity on the superhydrophobic and superoleophilic KHFM, and (2) to study the separation capacity of oil and water through the superhydrophobic and superoleophilic KHFM. For the absorption study, the oil–water mixture was prepared using 1.0 g *n*-hexane as oil and 3.0 g water which was dyed with methylene blue in a Petri dish. The absorption study was carried out by placing the superhydrophobic and superoleophilic KHFM prepared at various grafting coating cycles into the Petri dish containing the oil mixture. The oil absorption capacity (*Q*_a_) was recorded for 1 minute and calculated using the equation:2
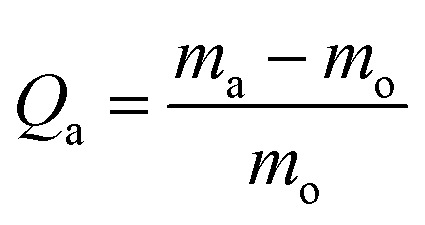
where *m*_a_ and *m*_o_ are the masses of the superhydrophobic and superoleophilic KHFM after and before adsorption, respectively.

For the separation study, the oil flux, *J*_O_ (L m^−2^ h^−1^) was calculated every 10 minutes based on the equation:3
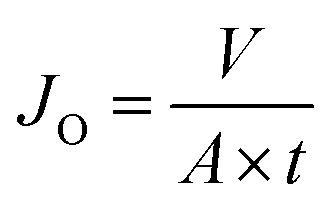
where *V* is the volume for oil permeate (L), *A* is the area for the superhydrophobic and superoleophilic KHFM, which is assembled into a module (*m*^2^), and *t* is time (h).

## 3. Results and discussions

**Fig. 4:**
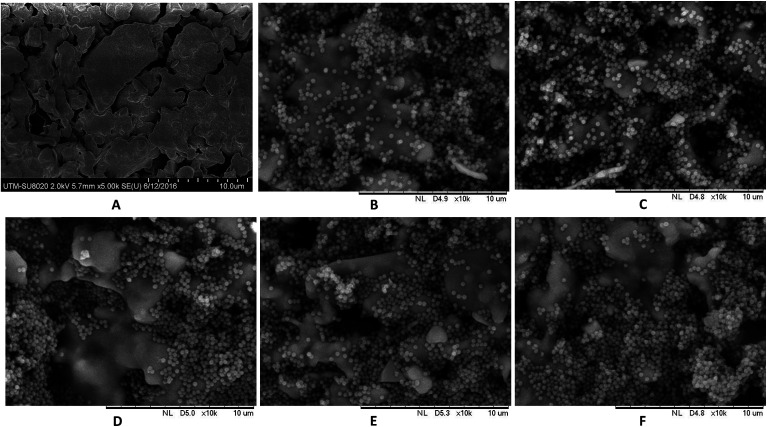
SEM surface images of (A) pristine KHFM and superhydrophobic/superoleophilic KHFM grafting at (B) 1 cycle, (C) 2 coating cycles, (D) 3 coating cycles, (E) 4 coating cycles, and (F) 5 coating cycles, of sol–gel.

**Fig. 5:**
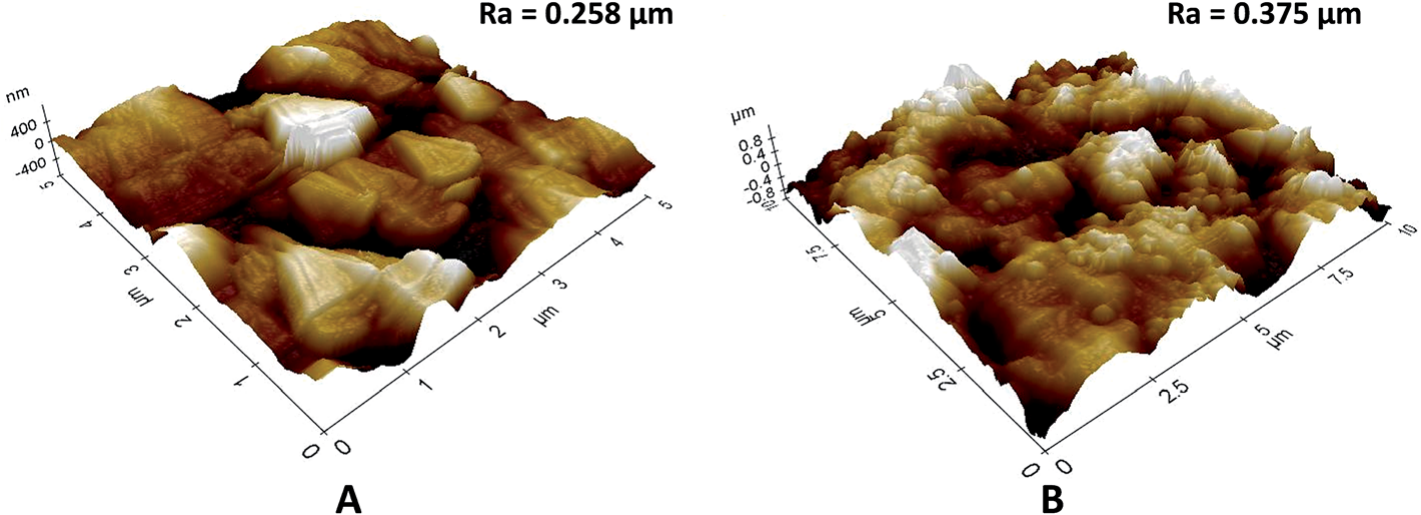
3D AFM images and surface roughness (*R*_a_) of (A) pristine KHFM, and (B) superhydrophobic/superoleophilic KHFM dip-coated at 2 coating cycles of sol–gel.

Fig. 4A shows SEM images of pristine KHFM surfaces, whereas Fig. 4B–F show the grafted KHFM surface by a dip coating technique with MTES and TEOS at various grafting coating cycles. As can be seen, the pristine KHFM surface shows a porous structure with a small neck growth mechanism that occurred between the kaolin particles during the sintering process. When grafting with MTES and TEOS, it can be observed that the KHFM surface was covered by a relatively small silica particle of nano size. As stated by Yang *et al.*,^16^ the size of the silica particles that can be obtained by this method was around 220 nm. It is worth mentioning that a close examination revealed the silica nanoparticles grafted at various grafting coating cycles depict a different homogeneity. When the KHFM was grafted for 1 coating cycle with MTES and TEOS, the silica nanoparticles covered the KHFM at certain places only. In fact, the silica nanoparticles were observed to agglomerate, and thereby create an inhomogeneous layer of silica nanoparticles. With an increase of the MTES and TEOS grafting coating cycles to 2 coating cycles, a relatively homogeneous layer of silica nanoparticles was created on the KHFM surface, resulting in a highly roughened KHFM surface (Fig. 5). Further increasing the grafting coating cycles from 2 coating cycles to 3, 4 and 5 coating cycles recreates the agglomeration mechanism that occurs when grafted for 1 coating cycle, which means the particles were making a layer that is not stable. This may happen due to the excessive silica nanoparticles reacting with the silica layer, creating a layering of another silica nanoparticle layer. Whereas, the inhomogeneous KHFM surface results from the weak bonding between the silica and the KHFM surface.

In order to study the surface roughness of KHFM before and after grafting, 3D AFM images of the pristine KHFM and grafted KHFM with 2 coating cycles, as this has a homogeneous structure (Fig. 5). With no particles grafted on the KHFM surface, the rougher KHFM surface appears with large particles with a surface roughness value of 0.258 μm (Fig. 5A) before grafting. When the silica nanoparticles were grafted onto the KHFM surface, the 3D AFM image (Fig. 5B) clearly shows the homogeneous distribution of silica particles on the KHFM surface. As a result, an increase in the surface roughness value was obtained after 2 coating cycles of grafting (0.375 μm). In fact, 3D AFM images of the grafted KHFM possessed more bright regions compare to pristine KHFM, indicating that most of the pores have been covered with the superhydrophobic and superoleophilic silica. As a matter of fact, Khayet and Matsuura^25^ stated that nodules are observed as bright high peaks in the 3D AFM images, whereas the membrane pores are seen as a dark region.

**Fig. 6:**
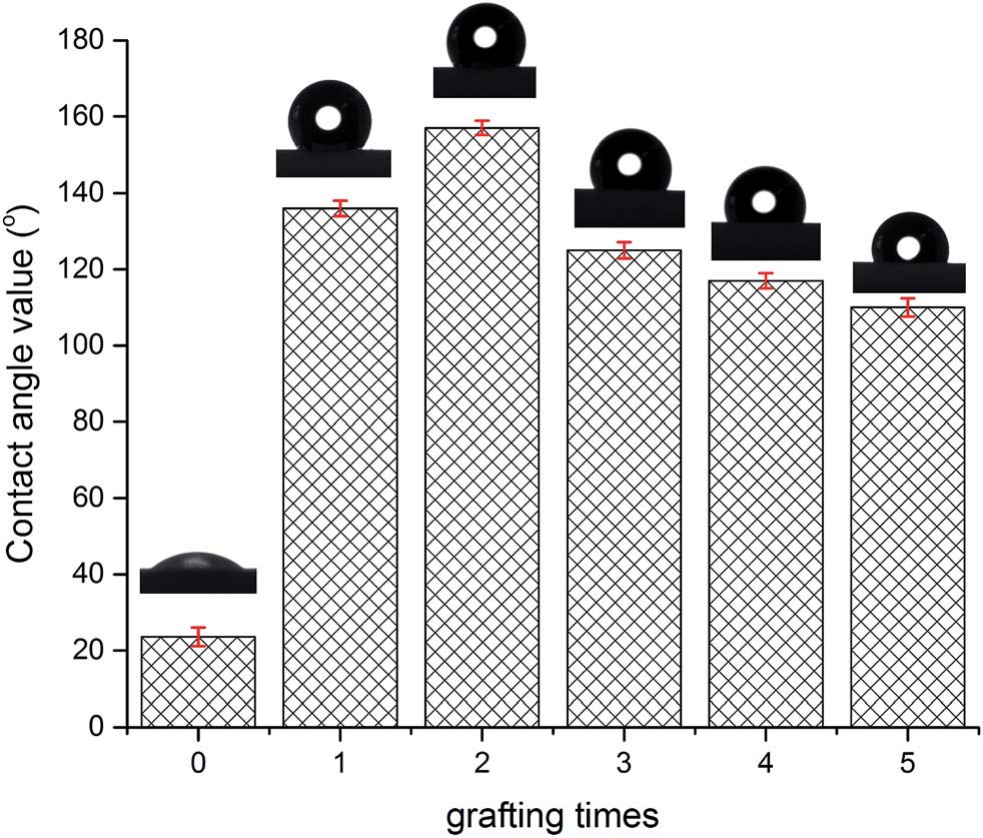
Water contact angle of pristine KHFM and superhydrophobic/superoleophilic KHFM.

**Fig. 7:**
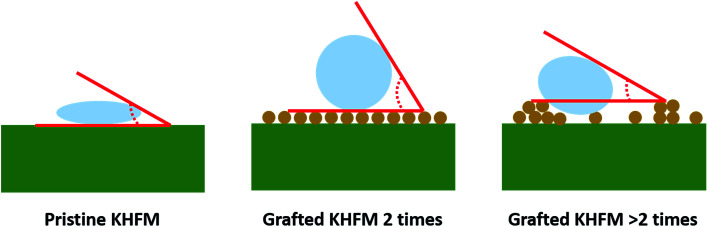
Schematic diagram of water droplets on pristine and grafted KHFM.

**Fig. 8:**
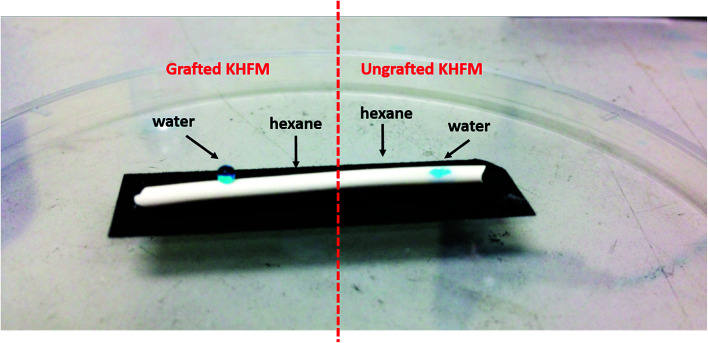
Photographic image of water and an *n*-hexane droplet on grafted and ungrafted KHFM.

Fig. 6 depicts the water contact angle value for the pristine and grafted KHFM. For the grafted KHFM, the contact angle was measured after the calcination process at 400 °C. In a similar trend to surface roughness, the contact angle value increased with the increase of grafting coating cycles from 1 to 2 coating cycles, and decreased when the grafting increased to 3, 4 and 5 coating cycles. The value of the contact angle for grafted KHFM was increased from 23.6° to 136° and 157°, and then decreased to 125°, 117° and 110°. Fig. 6 explains the effect of grafting time on the behaviour of a water droplet on the KHFM surface. A contact angle value of more than 90° exhibits hydrophobicity, while one of more than 150° was categorized as superhydrophobicity. Therefore, it was concluded that KHFM grafted for 2 coating cycles possessed a superhydrophobicity property. Hexane was used to measure the oleophilicity of the KHFM. As expected, a drop of hexane could be absorbed immediately once it touched the KHFM surface and penetrated into the sponge and finger-like pores of the KHFM within one second and thus no contact angle could be measured. A detailed description of the cross-sectional SEM image of the KHFM consisting of finger and sponge-like pores was published elsewhere.^23^ A photographic image (Fig. 8) of the KHFM also proved its superhydrophobicity and superoleophilicity for a blue water droplet dyed with methylene blue and sat on the KHFM surface with a nearly perfect spherical shape, while an *n*-hexane droplet wetted the KHFM surface. Before grafting, both of the droplets wetted the KHFM surface, indicating a superhydrophilic behaviour. Comparing with sponges or textiles,^26,27^ KHFM offers a green technology and excellent reusability (Fig. 7).

**Fig. 9:**
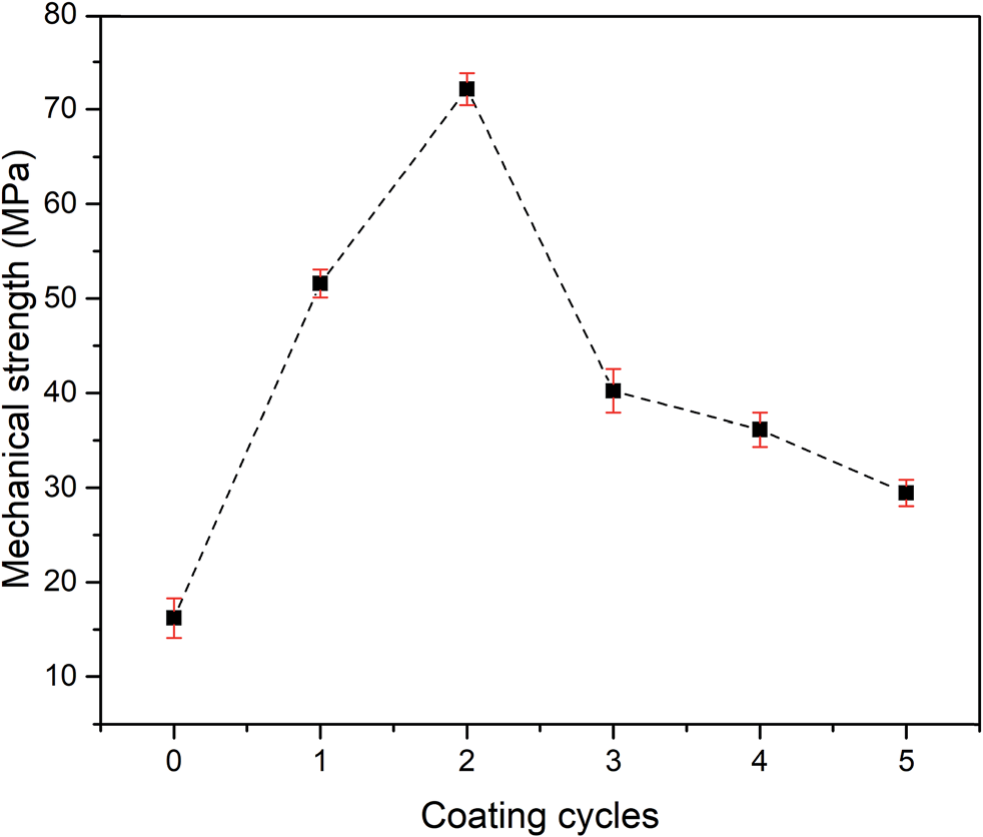
Mechanical strength of pristine KHFM and superhydrophobic/superoleophilic KHFM.

The result of a 3-point bending test, which was used to measure the mechanical strength of the pristine and grafted KHFM at various coating cycles with different homogeneities of silica nanoparticle distribution on the KHFM surface is shown in Fig. 9. The results showed that the mechanical strength increased with an increase of grafting coating cycle from the pristine membrane (0 coating cycle) (16.2 MPa) to 2 coating cycles (72.3 MPa). Then, the mechanical strength dropped significantly from 72.3 MPa to 29.5 MPa when the grafting coating cycles were increased from 2 to 5 cycles. However, it should be noted here that the mechanical strength of the pristine KHFM can be improved after the grafting process. In addition, enhancement of the membrane strength proved that no crack growth behaviour or membrane defect resulted from the grafting process. As stated by Miller *et al.*,^28^ a grafting or coating process provides mechanical integrity to the membrane to withstand the pressure required and may offer some physical coupling to the membrane due to the inherent roughness of the membrane surface, which obeys the roughness value obtained in this study (Fig. 5).

**Fig. 10:**
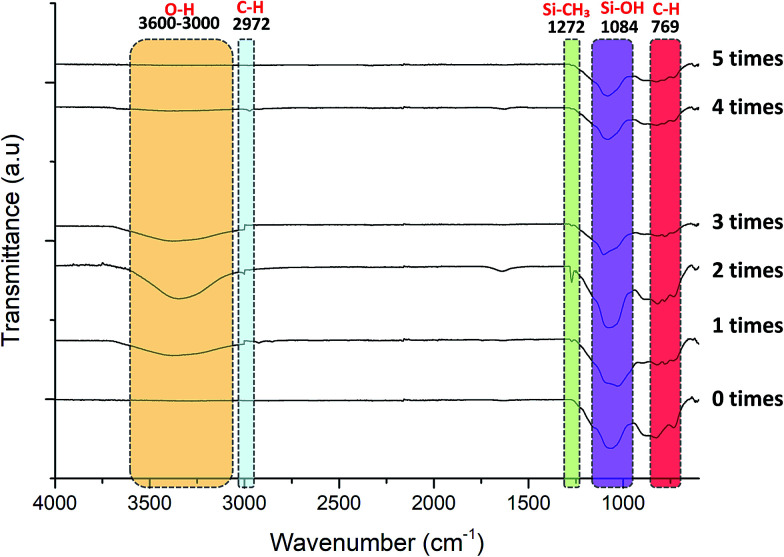
FTIR analysis of pristine KHFM and superhydrophobic/superoleophilic KHFM.

Fig. 10 exhibits the FTIR spectra of pristine and grafted KHFM at various grafting times. The stretching vibration bands of O–H (3600–3000 cm^−1^) appeared with a broad peak after KHFM was grafted with MTES and TEOS. The reason for the absence of this peak on the pristine membrane is due to the application of a high sintering temperature of 1300 °C which led to a dehydroxylation mechanism.^29^ Due to this, a hydrolysis step was required and done by immersing the KHFM into the mixture of ethanol and water to create OH bonding to react with OH from the sol–gel solution. This reaction creates C–H and Si–CH_3_ peaks at 2972 cm^−1^ and 1272 cm^−1^, respectively. Generally, KHFM grafted for 2 coating cycles was highly expected to have this peak compared to other KHFMs. A broad peak of the Si–OH band (1084 cm^−1^) increased with the increasing KHFM grafting number. The highest intensity of this band can be observed for KHFM grafted at 4 coating cycles, which reflects the expectation that should be induced by KHFM grafted at 2 coating cycles. Perhaps this is due to the inhomogeneous particle distribution of silica nanoparticles, as described previously. In conclusion, the FTIR analysis obtained in this work was in good agreement with the trend of adapted work by Yang *et al.*^16^

**Fig. 11:**
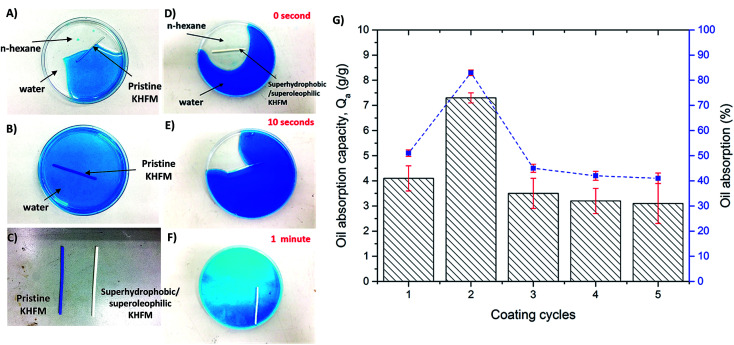
Photographic images of (A) pristine KHFM in the mixture of oil (*n*-hexane) and water (dyed with methylene blue); (B) in water; (C) Pristine KHFM and superhydrophobic/superoleophilic KHFM after testing in water; (D–F) superhydrophobic/superoleophilic KHFM for the removal of oil from the water through absorption; and (G) oil absorption capacity on superhydrophobic/superoleophilic KHFM.

Oil absorption on grafted superhydrophobic and superoleophilic KHFM at various coating times was investigated. In this work, *n*-hexane was used as the oil and water was dyed with methylene blue. As shown in Fig. 11A, the pristine KHFM was observed to absorb both water and *n*-hexane due to the absence of superhydrophobic properties. This result was further proved when the pristine membrane significantly sank in the water solution (Fig. 11B). Accordingly, Fig. 11C shows a comparison of the final pristine KHFM and the superhydrophobic/superoleophilic KHFM after testing with blue water. When a piece of superhydrophobic/superoleophilic KHFM was placed on the surface of an *n*-hexane–water mixture, the *n*-hexane was quickly absorbed by the grafted KHFM within a minute (Fig. 11D–F). The oil absorption capacity was calculated by measuring the weight of *n*-hexane that was captured by the grafted KHFM in 1 minute. Consequently, the percentages of oil absorption on the grafted KHFM at various grafting coating cycles were also recorded, as shown in Fig. 11G. As shown in Fig. 11G, the oil absorption capacity of the superhydrophobic/superoleophilic KHFM was in the range of 3.1–7.3 g g^−1^, which was comparable with that reported in the literature for superhydrophobic/superoleophilic sponges, such as magnetic silicon sponges (7–17 g g^−1^).^30^ As expected, the highest oil absorption was possessed by KHFM that grafted at 2 coating cycles with an absorption capacity value and oil absorption percentage of 7.3 g g^−1^ and 83%, respectively. Generally speaking, grafted KHFM at 2 coating cycles showed the most homogeneous structure of silica on the membrane surface (Fig. 4). Besides, the contact angle value also proved the ability of this grafted KHFM to reject water and absorb oil.

**Fig. 12:**
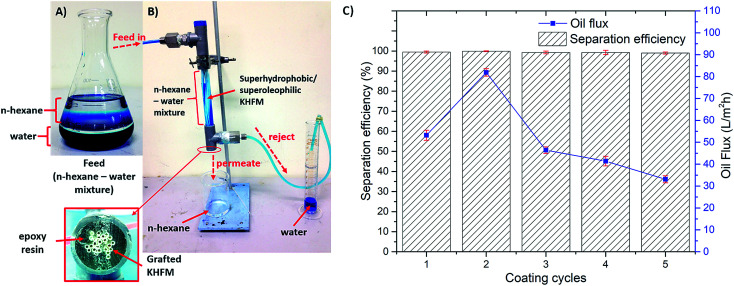
(A and B) Photographic image of oil–water mixture using hexane and water (dyed with methylene blue); (C) oil flux of superhydrophobic/superoleophilic KHFM.

In order to understand the oil–water separation efficiency, an in-house filtration system using gravity pressure was set-up as shown in Fig. 12(A and B). About 20 grafted KHFMs were potted using epoxy resin and assembled into a module. The module consisted 1 inlet stream and 2 outlet streams in which the oil–water mixture was sucked into the module, oil (*n*-hexane) permeated out through the KHFM bore and water (dyed with methylene blue) was rejected into a measuring cylinder. In this work, the volume of oil permeated with time was recorded as the oil flux to determine the efficiency of the KHFM separation. Fig. 12(C) shows the effect of grafting time on the permeated oil flux. As observed, increasing the grafting time from 1 coating cycle to 2 coating cycles leads to an increment of flux with time. Whereas, further increments from 2 coating cycles to 5 coating cycles resulted a similar trend to that of mechanical strength, surface roughness and contact angle. The highest flux was obtained by grafted KHFM at 2 coating cycles with a value of 102 L m^−2^ h^−1^ at the beginning of the filtration. This proved that the membrane performances were strongly influenced by membrane characteristics. However, in terms of mechanical strength, Paiman *et al.*^31^ stated that a higher mechanical strength led to a lower flux which was conversely obtained in this work. This was attributed to the superhydrophobic and superoleophilic behaviour of the KHFM that prevented pore blocking. In fact, the high oil flux was also influenced by the existence of an asymmetric structure, in which the grafted sponge-like structure surface absorbs the oil and repels the water, and, thereby, is captured in a finger-like structure, which leads to a higher flux.

Table 1 reports a comparison of the superhydrophobic/superoleophilic KHFM for oil/water separation with previous studies. As shown, there are three things that can be compared. In this study, a kaolin hollow fibre membrane was introduced and modified as a newly developed precursor for a superhydrophobic/superoleophilic application. As summarized, it was found that the newly developed superhydrophobic/superoleophilic KHFM in this study was comparable to other studies on application. Interestingly, the present study provides both absorption and filtration in a single step, thus solving the problem of filtration requirement. Although the oil absorption capacity was slightly higher compared with that of other work, it should be mentioned here that the separation efficiency was the highest (>99%). Therefore, it is suggested that the newly developed superhydrophobic/superoleophilic KHFM in this study possesses an excellent performance in separating oil and water at a lower cost and with green technology.

**Table 1:** Comparison of superhydrophobic/superoleophilic KHFM in this study with previous works

Type of precursors	Separation mechanism	Oil absorption capacity (g g^−1^)	Oil flux (L m^−2^ h^−1^)	Separation efficiency (%)	References
Sponge	Absorption	23–60	—	—	32
	Absorption	22–78	—	—	33
	Absorption–filtration with vacuum system	22–44	N/A	>99.5	34
Cotton fabric	Filtration	—	N/A	96–98	35
Copper mesh	Filtration	—	N/A	94–97	36
	Filtration	—	N/A	95	37
Stainless steel	Filtration	—	N/A	93–96	14
	Filtration	—	N/A	>90	13
Ceramic membrane	Absorption–filtration	3–7	33–82	>99%	This work

## 4. Conclusion

In this study, the superhydrophobic and superoleophilic kaolin hollow fibre membrane was successfully employed by grafting with silica sol–gel solution at grafting coating cycles ranging from 1 to 5 coating cycles for the treatment of oil removal from water. The results showed that the KHFM grafted at 2 coating cycles is the best superhydrophobic and superoleophilic for oil removal from water with a 99.9% rejection. In addition, this high removal was influenced by the highest water contact angle of 157° and a high mechanical strength of 72.33 MPa. Consequently, the KHFMs were easily assembled into a module and offered a high flux of 102 L m^−2^ h^−1^ at the pressure of gravity. Compared with other substrates, such as sponge, cotton and stainless steel, KHFM shows a green technology due to its degradable properties, cost effectiveness and long life time. A further study on the application of the newly developed superhydrophobic/superoleophilic KHFM towards real oil wastewater, such as palm oil mill effluent (POME), will be conducted.

## Conflicts of interest

There are no conflicts to declare.

## Supplementary Material
